# A New Herbal Formula, KSG-002, Suppresses Breast Cancer Growth and Metastasis by Targeting NF-****κ****B-Dependent TNF****α**** Production in Macrophages

**DOI:** 10.1155/2013/728258

**Published:** 2013-05-30

**Authors:** Sang-Mi Woo, Youn Kyung Choi, Sung-Gook Cho, Sunju Park, Seong-Gyu Ko

**Affiliations:** ^1^Laboratory of Clinical Biology and Pharmacogenomics, Department of Preventive Medicine, Kyung Hee University, 1 Hoegi, Seoul 130-701, Republic of Korea; ^2^Center for Clinical Research and Genomics, Department of Preventive Medicine, Kyung Hee University, Seoul 130-701, Republic of Korea

## Abstract

Tumor-associated macrophages (TAMs) in tumor microenvironment regulate cancer progression and metastases. In breast cancer, macrophage infiltration is correlated with a poor prognosis. While metastatic breast cancer is poor prognostic with a severe mortality, therapeutic options are still limited. In this study, we demonstrate that KSG-002, a new herbal composition of radices *Astragalus membranaceus* and *Angelica gigas*, suppresses breast cancer via inhibiting TAM recruitment. KSG-002, an extract of radices *Astragalus membranaceus* and *Angelica gigas* at 3 : 1 ratio, respectively, inhibited MDA-MB-231 xenograft tumor growth and pulmonary metastasis in nude mice, while KSG-001, another composition (1 : 1 ratio, *w/w*), enhanced tumor growth, angiogenesis, and pulmonary metastasis, *in vivo*. KSG-002 further decreased the infiltrated macrophage numbers in xenograft tumor cohorts. In Raw264.7 cells, KSG-002 but not KSG-001 inhibited cell proliferation and migration and reduced TNF-alpha (TNF**α**) production by inhibiting NF-**κ**B pathway. Furthermore, a combinatorial treatment of KSG-002 with TNF**α** inhibited a proliferation and migration of both MDA-MB-231 and Raw264.7 cells. Taken together, we conclude that KSG-002 suppresses breast cancer growth and metastasis through targeting NF-**κ**B-mediated TNF**α** production in macrophages.

## 1. Introduction

Tumor microenvironment is highly heterogeneous and dynamic in cell-to-cell communications, regulating tumor progression and metastases [1–4]. In tumor microenvironment, tumor-associated macrophages (TAMs) are known to regulate cancer growth and metastases through alterations of tumor cell proliferation, migration, invasion, angiogenesis, immunosuppression, and extracellular matrix alteration [1, 5–9]. Thus, TAMs can be one of cancer therapeutic targets. 

TNF-alpha (TNF*α*) has “yin and yang” roles in cancer development and metastases [10–12]. TNF*α* released from macrophages induces cancer cell proliferation and migration [13, 14]. TNF*α* expression is mainly regulated by NF-*κ*B signaling pathway [12, 15, 16]. Furthermore, TNF*α* activates NF-*κ*B pathway through TNF receptor I and II [12, 15, 16]. TNF receptors-mediated NF-*κ*B signaling involves IKK phosphorylation, I*κ*B phosphorylation and degradation, and NF-*κ*B nuclear translocation and transcriptional activation [12, 15–17]. Therefore, TNF*α* released from macrophages activates NF-*κ*B-mediated signaling pathway in cancer, resulting in cancer progression and metastasis [16]. Regarding the importance of TNF*α* in various diseases including cancer, therapeutic approaches to target TNF*α* have been applied, although agents recently developed have shown some side effects including tumor promotion [12, 18, 19].

Traditional herbal medicines have shown anti-cancer effects with no or less toxicity compared to other anti-cancer agents [8, 20–22]. Although anticancer effects of herbal extracts from radix *Angelica gigas* and/or radix *Astragalus membranaceus* have been revealed in different cancer cell types including hepatocellular carcinoma cells, cervical cancer, and prostate cancer cells [23–30], it is yet to be investigated whether those extracts affect highly metastatic cancers, especially breast cancer.

In this study, we developed new herbal formulae, KSG-001 and KSG-002, and identified that KSG-002 but not KSG-001 inhibits breast cancer growth, angiogenesis, and pulmonary metastasis by targeting TAMs. KSG-001 and KSG-002 were extracted from the herbal mixture of *Astragalus membranaceus* and *Angelica gigas* with 1 : 1 ratio (*w/w*) and 3 : 1 ratio (*w/w*), respectively. KSG-001 and KSG-002 showed no effect on a viability of different breast cancer cells and normal epithelial cells. However, KSG-002 but not KSG-001 effectively repressed TAM cell proliferation and migration with reductions of TNF*α* production by inhibiting NF-*κ*B pathway. Thus, our present study suggests that KSG-002 may be useful for treating cancer growth and metastases. 

## 2. Materials and Methods

### 2.1. Reagents and Cell Culture

KSG-001 and KSG-002, herbal mixtures of *Astragalus membranaceus* and *Angelica gigas* with 1 : 1 ratio (*w/w*) and 3 : 1 ratio (*w/w*), respectively, were extracted with 30% EtOH (Hanpoong Pharmaceutics, Jeonju, Republic of Korea). To verify quantities and qualities of mixtures, formononetin (for *Astragalus membranaceus*), decursin (for *Angelica gigas*), and nodakenin (for *Angelica gigas*) as the index compounds were analyzed with HPLC and UPLC (data not shown). MDA-MB-231 and BT-20 cells were cultured in DMEM supplemented with 10% fetal bovine serum (FBS) and 1% antibiotics. MCF-10A cells were maintained in DMEM/F12 medium with 5% horse serum, 20 ng/mL of EGF, 10 *μ*g/mL of insulin, 0.5 mg/mL of hydrocortisone, 100 ng/mL of cholera toxin, and 1% antibiotics. 

### 2.2. Cell Viability, Migration, Invasion, and Anchorage-Independent Assays

Cells were cultured in 96-well plates and treated with KSG-001 or KSG-002 for one to three days and subjected to the MTT assays. Assays were performed three times in sextuplicate. For the cell migration, cells cultured in 6-well plates were scratched and treated with extracts for 24 hours. Migrated cells were imaged using a camera connected to a microscope and counted. For the cell invasion, two-chamber assays were performed. Cells were cultured on the upper chamber and treated with extracts for 24 hours. Cells were stained with hematoxylin and eosin to capture photo images and counted. For the anchorage-independent assays, cells were mixed with soft agars and cultured for 15 days. Extracts were added every three days. All experiments were performed three times in triplicate. 

### 2.3. *In Vivo* Studies

Animal experiments were approved by Kyung Hee University Institutional Animal Care of Use Committee (KHU-IACUC). Nude mice were subcutaneously injected with 1 × 10^6^ MDA-MB-231 cells. When xenograft tumor volume reached 50 mm^3^, mice were randomly grouped (*n* = 5/group) and orally added with extracts (500 mg/kg/day). Xenograft tumor volume and mouse body weight were measured three times a week. At day 34 posttreatment, mice were sacrificed, and xenograft tumor bundles and other organs were fixed in 4% formaldehyde for further analyses. Tumor tissues were embedded in paraffin, dissected with 5 *μ*m, and stained with hematoxylin and eosin or appropriate antibodies. Anti-CD31 antibody (AbCam) was used for detecting angiogenic vessels. Anti-F4/80 antibody (AbD Serotec) for macrophages was kindly provided by Dr. Hyun-Soo Bae (Kyung Hee University, Seoul, Republic of Korea).

### 2.4. ELISA, Western Blot, and Luciferase Assays

Cells were treated with KSGs for 24 hours, and then media were gathered for ELISA. Levels of TNF*α*, IL-6, and IL-beta were analyzed with the appropriate ELISA kits (BD Biosciences, San Jose, CA, USA). For the western blot, 30 *μ*g of protein was loaded onto 6% to 12% SDS-PAGEs and transferred to PVDF membranes. Anti-p-NF-*κ*B, p-I*κ*B NF-*κ*B, p-IKK, iNOS, MMP-9, FAS, and COX-2 were purchased from Cell Signaling. Anti-Tubulin antibody was obtained from Sigma. Anti-I*κ*B antibody was purchased from Upstate Biotechnology. Anti-IKK antibody was obtained from Santa Cruz Biotechnology. For the luciferase assays, constructs for NF-*κ*B-luc (Stratagene) and TNF*α*-luc were kindly provided from Dr. Mingyao Liu (Texas A&M University, Houston, TX, USA), and plasmids for IKK and p65 were received from Dr. Barat B. Aggarwal (University of Texas MD Anderson Cancer Center, Houston, TX, USA). Raw264.7 cells were transfected with the appropriate plasmids for 24 hours and then treated with extracts or not for another 6 hours. The luciferase activities were done by manufacturer's manuals (Promega). 

### 2.5. Statistics

Statistical significances were calculated by the Student's *t*-test or one-way ANOVA, as appropriate. *P* value less than 0.05 was considered statistically significant. 

## 3. Results

### 3.1. KSG-002 Suppresses TNBC Tumor Growth and Metastasis *In Vivo*

**Figure 1:**
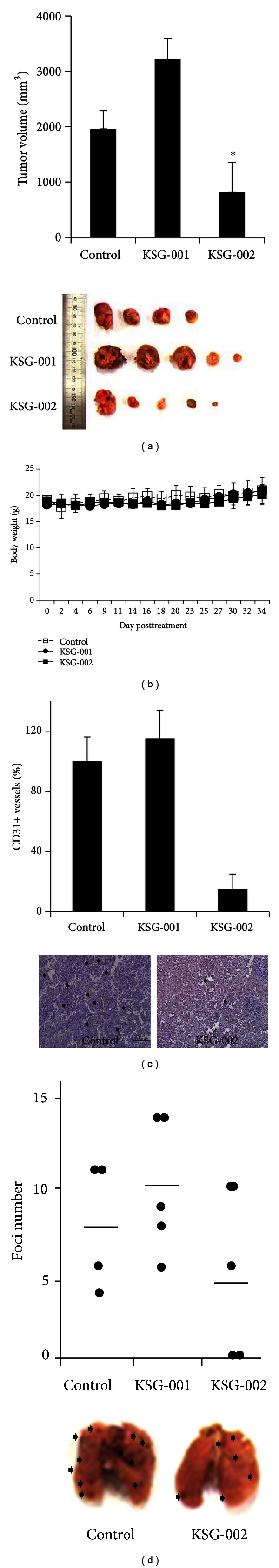
KSG-002 suppresses xenograft tumor growth and metastasis. (a) 1 × 10^6^ MDA-MB-231 cells were injected subcutaneously into nude mice and were orally added with the extract (500 mg/kg/d) indicated. Xenograft tumor volumes at day 34 posttreatment were presented as means ± standard deviations. *P < 0.05. Images show tumors xenografted. (b) Body weights were measured three times a week. (c) CD31-positive vessels in tumor cohorts were counted. Bars indicate means ± standard deviations. *P < 0.05. Representative images for anti-CD-31 antibody staining. Arrows indicate CD31-positive vessels. (d) Metastatic tumor foci at the lung were counted. Dots and bars indicate individual numbers and means, respectively. Arrows indicate metastatic foci.

To investigate effects of KSGs on breast tumor growth and metastases *in vivo*, nude mice (*nu/nu*) were subcutaneously injected with highly metastatic MDA-MB-231 cells, when tumor volume reached about 50 mm^3^, orally administrated with KSG-001 (500 mg/kg/d) or KSG-002 (500 mg/kg/d). When xenograft tumor volume was measured three times a week, KSG-002, compared to control, suppressed xenograft tumor growth. However, KSG-001, compared to control, rather increased tumor volume. At day 34 posttreatment, the average volume of xenograft tumors in control (*n* = 4), KSG-001 (*n* = 5), and KSG-002 (*n* = 5) was approximately 1960 mm^3^, 3220 mm^3^, and 815 mm^3^, respectively (Figure 1(a)). However, both KSG-001 and KSG-002 did not affect body weights (Figure 1(b)).

When tumor angiogenic vessels were examined with anti-CD31 antibody staining, KSG-002, compare to control or KSG-001, reduced angiogenic vessel numbers (Figure 1(c)). As KSG-002 appeared to inhibit tumor progression and angiogenesis, we further examined whether KSG-002 inhibited tumor metastasis. Mice were sacrificed at day 34 posttreatment, and lungs from each group were dissected for investigating metastatic colony formation at the lungs. When metastatic foci at lung were counted, KSG-002 (avg. = 5.2), compared to control (avg. = 8.25), appeared to reduce metastatic colony numbers while it was not statistically significant, and KSG-001 (avg. = 10.2) increased those numbers (Figure 1(d)). 

### 3.2. KSG-002 Does Not Affect Tumor Cell Growth, Migration, Invasion, and Anchorage-Independent Growth

**Figure 2:**
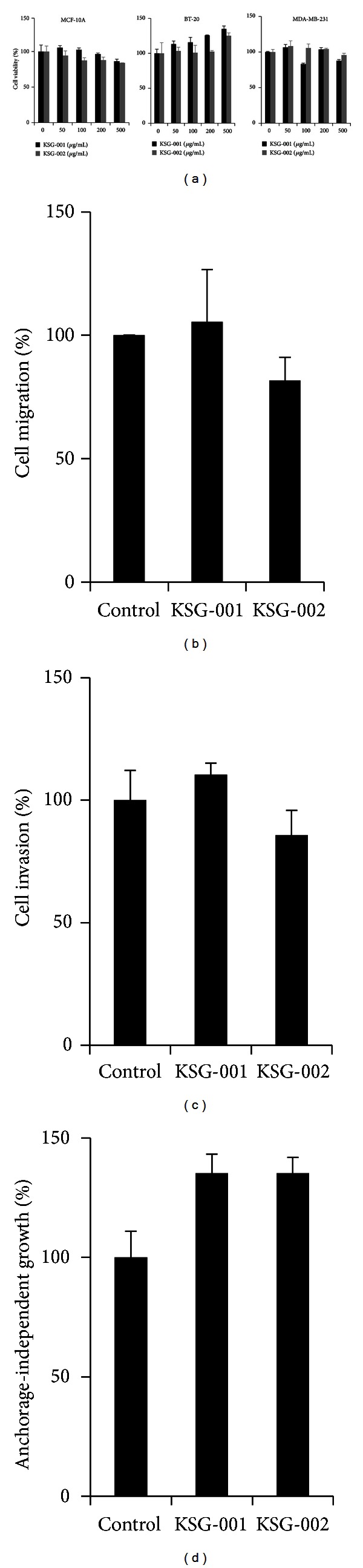
KSGs do not affect viabilities of both normal and tumor cells. (a) Cell viabilities of different cell types. Experiments were repeated three times in triplicate. Representative data show the mean and standard deviation. (b) Migration assays. MDA-MB-231 cells were scratched and treated with either KSG-001 or KSG-002 at 50 *μ*g/mL for 24 hours. Cells migrated from the wound edge were counted. Experiments were performed three times. Representative data show the means ± standard deviations. (c) Invasion assays. MDA-MB-231 cells cultured on the upper chamber precoated with Matrigels were added with serum-reduced medium, and the bottom chamber was added with complete medium. KSG-001 or KSG-002 was added on the upper chamber. Invaded cells were stained with crystal violet and counted. Experiments were repeated three times. Data indicate means ± standard deviations. (d) Anchorage-independent growth assays. MDA-MB-231 cells were cultured in soft agars for 15 days and stained with crystal violet. Experiments were performed in triplicate. Data represent means ± standard deviations.

As KSGs affected tumor growth *in vivo*, we next examined effects of KSGs on tumor cell viability. MCF-10A, BT-20, and MDA-MB-231 cells were treated with different doses of either KSG-001 or KSG-002 for 72 hours and subjected to MTT assays. As results, KSG-001 and KSG-002 did not significantly affect viabilities of normal and tumor cells (Figure 2(a)). 

We next examined breast cancer cell migration, invasion, and anchorage-independent growth. Both KSG-001 and KSG-002 at 50 *μ*g/mL did not affect MDA-MB-231 cell migration, invasion, and anchorage-independent growth (Figures 2(b)–2(d)). Thus, KSG-001 or KSG-002 seems not to affect breast cancer cells directly.

### 3.3. KSG-002 Inhibits Macrophage Infiltration

**Figure 3:**
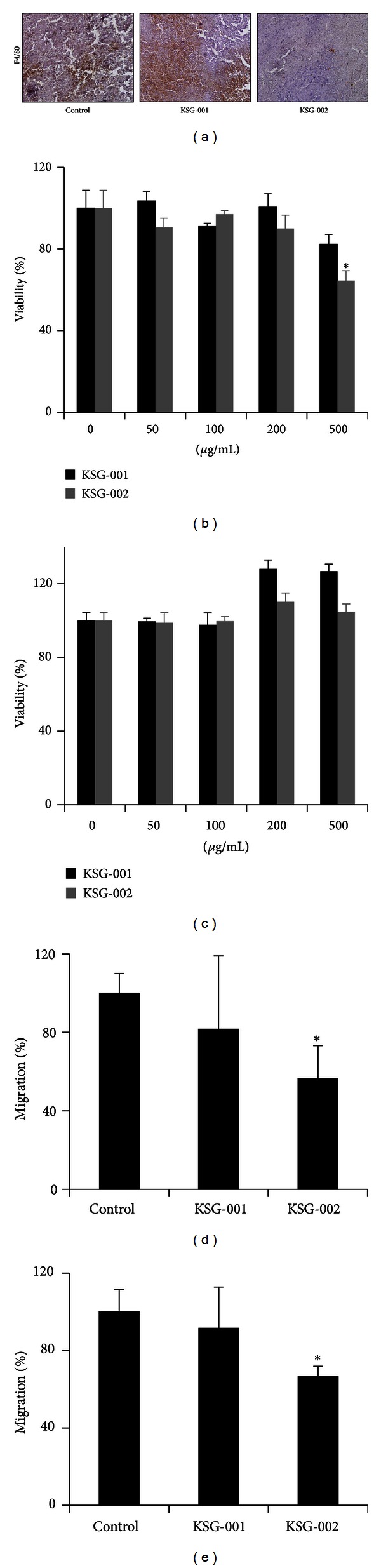
KSG-002 inhibits macrophage infiltration. (a) Infiltrating macrophage numbers in tumor cohorts. Xenograft tumor tissues were stained with anti-F4/80 antibody. 20x objectives. (b) Raw264.7 cell viability. Cells were treated with each extract at different concentrations for 48 hours and subjected to MTT assays. Experiments were performed three times in triplicate. Representative data indicate mean ± standard deviation. *P < 0.05 (versus control). (c) Raw264.7 cells were cultured in MDA-MB-231-conditioned medium and treated with KSG-001 or KSG-002 for 48 hours. Experiments were performed three times. Data indicate mean ± standard deviation. (d) Raw264.7 cells were cultured on the upper chamber and treated with KSG-001 or KSG-002. The bottom chamber was added with MDA-MB-231 medium. Raw264.7 cells migrated were stained with crystal violet and counted. Experiments were performed three times. Data show means ± standard deviations. *P < 0.05. (e) MDA-MB-231 cells were cultured on the upper chamber, and the bottom chamber was filled with Raw264.7 media that were obtained from Raw 264.7 cells treated with KSG-001 or KSG-002 for 24 hours. Cells were stained with crystal violet and counted. Experiments were done three times. Data indicate mean ± standard deviation. *P < 0.05 (versus control).

KSG-002, but not KSG-001, inhibited breast cancer growth *in vivo*. However, KSG-002 did not affect breast cancer cell viability, migration, invasiveness, and anchorage-independent growth *in vitro* (Figures 1 and 2). Tumor microenvironment includes tumor and stromal cells, of which crosstalks are crucial for tumor progression and metastases [1–3]. As TAMs are known to regulate tumor progression even in breast cancer [1, 6, 7], we next examined whether KSG-002 affects TAMs. When xenograft tumor tissues were stained with macrophage-specific monoclonal anti-F4/80 antibody, KSG-002, compared to either control or KSG-001, reduced the number of TAMs in tumor tissues (Figure 3(a)). 

Thus, we further examined whether KSG-002 directly affects macrophages. When mouse macrophagic Raw264.7 cells were treated with different doses of either KSG-001 or KSG-002 for 48 hours, KSG-002 reduced Raw264.7 cell viability at 500 *μ*g/mL by approximately 37.5% (Figure 3(b)). Nevertheless, KSG-002 and KSG-001 did not affect a viability of Raw264.7 cells, when Raw264.7 cells cultured in conditioned medium from MDA-MB-231 cells were treated with different concentrations of KSG-001 or KSG-002 for 48 hours (Figure 3(c)). 

Next, we examined whether KSG-002 affects chemotactic migration of macrophages. Raw264.7 cells on the upper chamber were treated with either KSG-001 or KSG-002, and MDA-MB-231 medium was added on the bottom chamber as a chemoattractant. KSG-002 at 500 *μ*g/mL inhibited Raw264.7 cell migration while KSG-001 at 500 *μ*g/mL did not affect it (Figure 3(d)), indicating that KSG-002 blocks macrophage infiltration in tumor bundle. 

We further examined whether KSG-002 affects a chemotactic migration of tumor cells. When Raw264.7 cells were treated with either KSG-001 or KSG-002 for 24 hours and MDA-MB-231 cells were cultured in Raw264.7-conditioned medium, KSG-002-treated Raw264.7 medium blocked chemotactic migration of MDA-MB-231 by approximately 37% (Figure 3(e)). Data indicate that KSG-002 may regulate products secreted from macrophages. 

### 3.4. KSG-002 Reduces TNF*α* Production of TAMs by Inhibiting NF-*κ*B Phosphorylation

**Figure 4:**
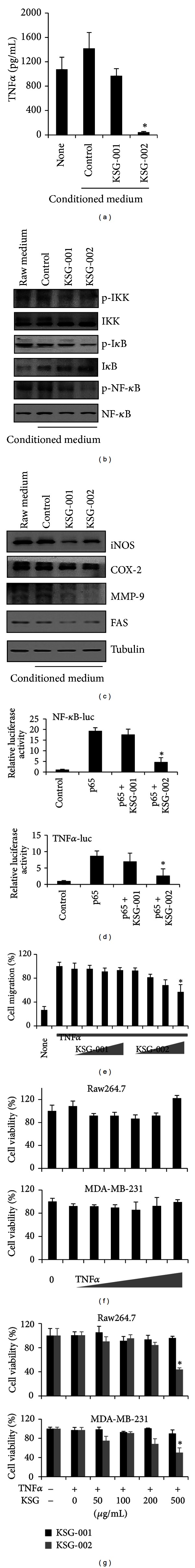
KSG-002 inhibits TNF*α* production. (a) TNF*α* levels were analyzed with ELISA assays. Experiments were performed in triplicate. Bars indicate means ± standard deviations. *P < 0.05. (b) KSG-002 inhibits NF-*κ*B signaling pathway. Western blots were performed with the appropriate antibodies. (c) KSG-002 inhibits expressions of NF-*κ*B-dependent genes. Western blots were done using antibodies for proteins indicated. Tubulin was used for the loading control. (d) The luciferase activities. Raw264.7 cells were transfected with p65 plasmid and NF-*κ*B-luc (top) or TNF*α*-luc (bottom) plasmid for 24 hours and then treated with KSGs (200 *μ*g/mL) for another 6 hours. The experiments were performed in triplicate. *P < 0.05. (e) MDA-MB-231 cells were scratched and treated with 100 ng/mL of TNF*α* alone or cotreated with TNF*α* (10^2^ ng/mL, colored in gray) and KSGs for 24 hours. Right triangles indicate different concentrations of KSGs (50, 100, 200, and 500 *μ*g/mL). Migrating cell numbers were counted, and data were presented as means ± standard deviations. Experiments were performed three times. *P < 0.05, TNF alone versus TNF*α* plus KSG-002. (f and g) Cells indicated were treated with TNF*α* alone (f) or together with KSGs (g) for 48 hours and subjected to MTT assays. Right triangles indicate different concentrations of TNF*α* (10^−3^, 10^−2^, 10^−1^, 1, 10, and 10^2^ ng/mL). For the combination, 10^2^ ng/mL of TNF*α* was treated with different concentrations of KSGs as indicated. Experiments were performed three times in triplicate. Representative data show mean ± standard deviation. *P < 0.05.

TNF*α* released from macrophages activates NF-*κ*B-mediated signaling pathway in cancer, resulting in cancer progression and metastasis [16]. To examine whether KSGs affect macrophagic TNF*α*, Raw264.7 cells were cultured with conditional medium from MDA-MB-231 cells, and media from Raw264.7 were subjected to ELISA assays. KSG-002, but not KSG-001, reduced TNF*α* level produced by Raw264.7 cells by approximately 86.5% (Figure 4(a)). 

As TNF*α* expression is regulated by NF-*κ*B signaling pathway, we further examined NF-*κ*B pathway molecules. Raw264.7 cells were cultured in conditioned medium from MDA-MB-231 cells and treated with 500 *μ*g/mL of either KSG-001 or KSG-002 for 24 hours. Both KSG-001 and KSG-002 reduced phosphorylation of IKK, I*κ*B, and NF-*κ*B (Figure 4(b)). Furthermore, both extracts inhibited expression levels of NF-*κ*B-dependent genes such as iNOS, COX2, Fas, and MMP-9 (Figure 4(c)). Thus, we further examined whether KSGs affect NF-*κ*B transcriptional activity. In the luciferase assays, while KSG-002 inhibited NF-*κ*B transcriptional activity in cells overexpressed with p65 and NF-*κ*B-luc, KSG-001 did not affect it (Figure 4(d), top). To confirm this inhibitory effect of KSG-002 on NF-*κ*B transcriptional activity, we performed the luciferase assays with TNF*α* promoter construct (TNF*α*-luc). In Raw264.7 cells transfected with TNF*α*-luc and p65, KSG-002 but not KSG-001 decreased NF-*κ*B-dependent TNF*α* promoter activity (Figure 4(d), bottom). Thus, our data indicate that KSG-002 selectively inhibits NF-*κ*B-dependent TNF*α* production.

Hence, we further examined whether KSG-002 affects macrophagic TNF*α*-induced breast cancer cell migration. MDA-MB-231 cells were treated with TNF*α* alone or cotreated with TNF*α* and KSGs for 24 hours. KSG-002 at 500 *μ*g/mL inhibited TNF*α*-induced MDA-MB-231 cell migration by approximately 43%, while KSG-001 did not affect it (Figure 4(e)). We further examined whether KSG-002 affects the viability of either tumor cells or macrophages in TNF*α*-confluent tumor microenvironment. Whereas TNF*α* alone did not affect cell viability of either Raw264.7 or MDA-MB-231 cells (Figure 4(f)), KSG-002 plus TNF*α* reduced the viabilities of both cell types by approximately 60% (Raw264.7) and 50% (MDA-MB-231), respectively (Figure 4(g)). Those data indicate that KSG-002 inhibits both cell proliferation and migration by enhancing cellular sensitivity to TNF*α*. 

## 4. Discussion

Tumor microenvironment, which consists of tumor cells and stromal cells such as vessel cells, fibroblasts, and immune cells, is highly heterogeneous and complex in inter-/intracellular mechanisms [1–3]. Macrophages in tumor microenvironment promote tumor progression [2, 4–7], and a number of infiltrated macrophages are an independent prognostic marker [31–33]. Triple negative breast cancer (TNBC) is highly metastatic and associated with a poor prognosis [34–36]. Nevertheless, therapeutic agents for TNBC have been yet developed [37–39]. In this study, we developed KSG-001 and KSG-002, new herbal formulae with radices *Astragalus membranaceus* and *Angelica gigas*, and found that KSG-002, but not KSG-001, suppressed MDA-MB-231 (defined as TNBC cells) growth and metastasis by inhibiting macrophagic TNF*α* production. 

**Figure 5:**
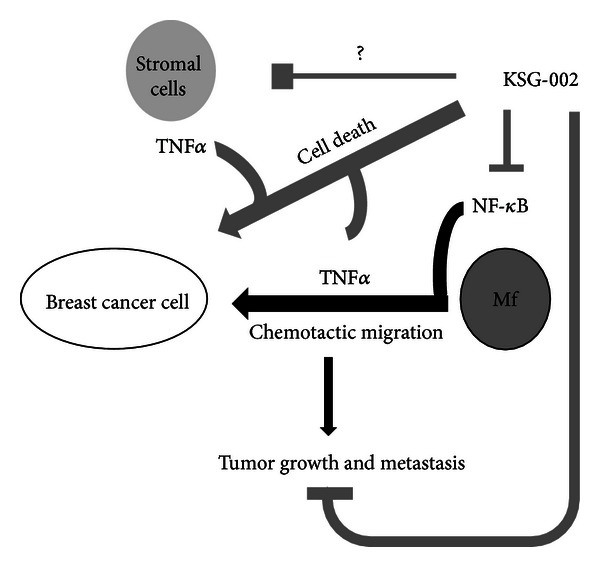
Schematic cartoon of KSG-002 effects on TNBC microenvironment. KSG-002 inhibits breast tumor growth and metastasis by blocking NF-*κ*B-dependent TNF*α* production in macrophages. KSG-002 in our study inhibited TNF*α* production from macrophages through blocking NF-*κ*B pathway, resulting in the suppression of tumor cell growth and metastasis. As TNF*α* is abundant in tumor microenvironment and produced from different types of cells, it still remains to elucidate whether KSG-002 targets other cell types producing TNF*α*. Interestingly, KSG-002 with TNF*α* caused breast cancer cell death, indicating that KSG-002 may sensitize cells to TNF*α*. Thus, KSG-002 seems to have pleiotropic roles in tumor microenvironment, while our present data demonstrate its role against macrophagic TNF*α* production in metastatic breast cancer microenvironment.

As mentioned, infiltrating TAM numbers is associated with a poor prognosis [31–33]. TAMs release different cytokines and regulate tumor cell growth, migration, and invasion [4, 6]. TNF*α* produced by TAMs is known to promote cancer growth [12, 15]. A number of F4/80-positive TAMs were reduced in tumor burdens of KSG-002-treated groups in our *in vivo* study. Furthermore, KSG-002 reduced TNF*α* production from TAMs and repressed cell growth and chemotactic migration of both TAMs and MDA-MB-231 cells. Consistently, we found a reduction of TNF*α* level in the blood of KSG-002-treated mice, while levels of IL-1β and IL-6 were not altered (data not shown). TNF*α* production involves NF-*κ*B transcriptional activation via phosphorylation of I*κ*B and NF-*κ*B [16]. Thus, an inhibition of I*κ*B/NF-*κ*B phosphorylation by KSG-002 may result in a reduction of TNF*α* production from TAMs, suppressing breast cancer growth and metastasis (Figure 5). We found that KSG-002 selectively repressed NF-*κ*B-dependent regulation of TNF*α* expression, while both KSG-001 and KSG-002 affected expression levels of NF-*κ*B-dependent genes such as iNOS, COX-2, MMP-9, and FAS. In addition, we found that KSG-002 inhibited JAK1/2 phosphorylation but not phosphorylation of STAT3 (data not shown). Thus, it remains to examine crosstalks between JAK/STAT pathway and NF-*κ*B pathway in KSG-002 effect on tumor microenvironment. In addition, while an extract from *Angelica gigas* showed cytotoxicity in rat intestinal epithelial (RIE) cells, KSG-002, a mixture of extracts from *Astragalus membranaceus* and *Angelica gigas* with 3 : 1 ratio, did not affect RIE cell viability (data not shown). In addition, KSG-001 rather increased tumor size. Thus, KSG-002 seems to have both the efficacy and safety, when two components are extracted with the appropriate ratio (3 : 1 in our study).

As KSG-002 is herbal extracts containing multiple components, it is possible that KSG-002 may target other stromal cells such as vessel cells, fibroblasts, and immune cells. In our histochemistry data, CD-31-positive vessel numbers were reduced by KSG-002. While TAMs regulate tumor angiogenesis [40, 41], it is also plausible that KSG-002 may directly target endothelial cells. Thus, it still remains to decipher multitarget effects of KSG-002 in tumor microenvironment. In addition, it is also worth to investigate effects of KSG-002 on different cancer types as well as different breast cancer subtypes. In this study, we focused on roles of KSG-002 in breast cancer cells, especially highly metastatic breast cancer cells, and found that the combination of KSG-002 with TNF*α* reduced MDA-MB-231 cell viability, while KSG-002 or TNF*α* alone did not affect it. Thus, further researches for KSG-002 effects on hormone-positive or HER2-positive breast cancer progression will provide evidence on anticancer effects of KSG-002 in different types of breast cancer cells, in depth and breadth. Furthermore, KSG-002 can be applied in different cancer cell types that are highly metastatic, as TNF*α* may be plentiful in metastatic tumor cohorts. When Raw264.7 cells were treated with KSG-002, cell viability was reduced. Likewise, KSG-002 decreased viabilities of Raw264.7 and MDA-MB-231 cells when cotreated with TNF*α*. However, KSG-002 did not affect the viability of Raw264.7 cells cultured in tumor medium. Thus, certain factors released from tumor cells may negatively affect KSG-002 effect, although it remains yet to know factors or mechanisms by which KSG-002 effect is abolished when macrophages and tumor cells are cocultured. 

In this study, we demonstrate that KSG-002, a novel herbal formula, suppresses highly metastatic breast cancer cells by inhibiting NF-*κ*B-mediated TNF*α* production from TAMs. Therapeutic agents for highly metastatic TNBC are yet to be developed, and regimens used for other cancer types are not working well. Traditional herbal medicines have been used for anticancer therapy, since those compared to anticancer drugs used in present have no or less side effect. While we need more efforts to determine KSG-002 functions in cancer disease, our present study for KSG-002 effect on breast cancer provides knowledge for therapeutic options.

## 5. Conclusion

In this study, we demonstrate that a new herbal formula, KSG-002, suppresses breast cancer growth and metastasis by blocking NF-*κ*B-dependent TNF*α* production from macrophages. Although we still make efforts to clearly understand its inhibitory roles in different cancer types and biological mechanisms in cellular and physiological levels, our present data suggest that KSG-002 may be useful for treating highly metastatic breast cancer.
